# Editorial: Insights in pharmacology of anti‐cancer drugs: 2021

**DOI:** 10.3389/fphar.2022.1062640

**Published:** 2022-10-21

**Authors:** Patricia Sancho, Olivier Feron

**Affiliations:** ^1^ IIS Aragón, Hospital Universitario Miguel Servet, Zaragoza, Spain; ^2^ Pole of Pharmacology and Therapeutics, Institut de Recherche Expérimentale et Clinique (IREC), UCLouvain, Brussels, Belgium; ^3^ Walloon Excellence in Life Sciences and Biotechnology (WELBIO), Wavre, Belgium

**Keywords:** drug repurposing, natural compounds, drug resistance, biomarkers, pharmacology

Challenges in the field of the pharmacology of anticancer drugs are numerous. One of them directly derives from the breakthrough development of anticancer immunotherapy. The enthusiasm around immune checkpoint blockers and CAR-T cells may indeed lead to neglect other strategies in the fight against cancer. This Research Topic is intended to gather some inspiring experimental research for future drug development.

Although there are thousands of publications every month dealing with potential anticancer activity of natural compounds, the translation of these findings in the clinic is limited. The field probably needs additional directions to take advantage of this tremendous effort to propose new drug candidates. Drug repurposing is of interest in this context. The identification of new indications for existing anticancer compounds may indeed offer advantages over traditional drug development including cost-effectiveness and reduced timelines. Repurposing of approved anticancer drugs becomes especially attractive when considering rare cancers or cancers with poor outcomes based on currently available treatments. Glioblastoma is one of these cancers with very limited therapeutic options. This RT contains reports showing treatment of glioma/glioblastoma with drugs (including natural products) previously used to treat other cancers or even pathologies not related to cancer. A brief summary of these studies are detailed here below.

PBI-05204, a chemically defined extract from *Nerium oleander* containing the cardiac glycoside oleandrin, has previously shown activity against different cancer types in preclinical studies *in vitro* and *in vivo*. Interestingly, this compound has been tested in Phase I and II clinical trials for patients with solid cancers including pancreatic cancer, proving to be safe for oral administration. The study by Colapietro et al. now demonstrates that treatment with PBI-05204 sensitizes glioblastoma cells to radiation therapy, by inducing apoptosis subsequent to DNA repair inhibition. Importantly, the combination of irradiation and PBI-05204 shows promising results in murine models of glioblastoma, inhibiting tumor progression and significantly improving survival of animals with subcutaneous and orthotopic GBM tumors.

Brusatol is an herbal extract isolated from the from the seeds of *Brucea sumatrana* that has been used in traditional Chinese medicine for treatment of infectious diseases. Interestingly, brusatol has shown anti-cancer activity in different preclinical models of leukemia as well as lung and gastrointestinal cancers. The study by Dai et al. builds on a previous observation suggesting that this compound may also be effective in GBM. Using preclinical models of GBM and primary human glioblastoma cells, these authors identify extracellular matrix protein 1 (ECM1) as the main factor implicated in the molecular response to brusatol.

Another article from this RT explores the use of a tyrosine kinase inhibitor trametinib (not derived from plant extracts) to treat the same cancer type (ie, glioma). This study underlies another attractive direction for the future of anticancer pharmacology, that is to explore the efficacy of existing therapies in other cancers than those for which approval was initially provided. Trametinib is a MEK1/2 inhibitor approved by the FDA for treatment of several cancer types. This compound was initially approved for treatment of BRAF V600E metastatic melanomas, showing survival improvement of patients with brain metastases. The work by Gao et al. documents the use of trametinib to treat glioma and identifies the metabolic rewiring of cancer cells *via* modulation of the PKM2/c-myc pathway as one of the mechanisms of action of this MEK inhibitor.

Besides drug repurposing and/or the targeting of the deadliest cancers, topics of interest also include pathways that are induced by anticancer drugs and that would gain in being inhibited to prevent the occurrence of resistance. The development of new anticancer drugs with a large panel of potential effects such as inhibitors of histone deacetylases (HDAC) and sirtuins has recently led to the identification of new targets to reinforce the efficacy of these non-selective drugs. Salazar-Gonzalez et al. report how the above modulators of protein acetylation eventually impact on the acetylation and activity of arylamine-N-acetyltransferase (NAT1). In another article, Guo et al. illustrate how resistance to lenvatinib in hepatocarcinoma is influenced by tRNAs which in turn impact on KRAS translation. Importantly, these authors identify YRDC as an attractive target for a combo treatment or at least as a biomarker to anticipate drug sensitivity.

Finally, a last article summarizes the recent development in the use of imaging biomarkers to determine the extent of tumor hypoxia, and thereby to optimize the use of various anticancer drugs. Although the tumor microenvironment is nowadays recognized as a major actor in the response to treatments, the implementation of this knowledge in the clinic has suffered from the paucity of robust and valuable biomarkers. Low pO_2_ in tumors was historically reported as a limiting factor in the success of radio- and chemotherapy but more recently the efficacy of targeted therapies as well as immune checkpoint inhibitors was also reported to be directly influenced by the extent of hypoxia. Gallez reviews the most exciting preclinical and clinical strategies to track tumor hypoxia as they have emerged in the last decade.

This special edition Research Topic puts a spotlight on ongoing progresses in the pharmacological field of anti-cancer drugs. May some of the above listed studies provide direction and guidance to researchers in the field.

